# Study on Seepage Characteristics of Grouting Slurry for Water-Absorbing Mudstone with Rough Fissure

**DOI:** 10.3390/ma17040784

**Published:** 2024-02-06

**Authors:** Zhe Chen, Yue-Jin Zhou, Lei-Ming Zhang, Yu-Nong Xu

**Affiliations:** 1 State Key Laboratory of Intelligent Construction and Healthy Operation and Maintenance of Deep Underground Engineering, China University of Mining and Technology, Xuzhou 221116, China; ts22030001a31@cumt.edu.cn (Z.C.); ts19030027a31@cumt.edu.cn (L.-M.Z.); xuyunong@cumt.edu.cn (Y.-N.X.); 2 School of Mechanics and Civil Engineering, China University of Mining and Technology, Xuzhou 221116, China

**Keywords:** mudstone, roughness, fissure grouting, water loss effect of slurry

## Abstract

Based on the computed tomography scanning, which abbreviation is CT scanning, and fractal theory, geometric parameters of mudstone fissures are obtained. The physical model of a single fissured channel is obtained in combination with Barton standard curves and 3D printing technology, and similar materials of mudstone are developed based on the water absorption of natural mudstone to prepare single fissured water-absorbing grouting test blocks with different roughness levels for the grouting simulation testing. By analyzing the viscosity change characteristics of grouting slurry before and after grouting, the seepage characteristics of the grouting slurry in the rough fissures of the water-absorbing mudstone are revealed. The results show that when the roughness is small, the grouting slurry will have an obvious water loss effect after passing through mudstone fissures. However, with the flow of the slurry, the water loss effect of the subsequent grouting slurry will be weakened. For fissures with high roughness, the water absorption properties of the rough surfaces and the walls of the mudstone fissures work together, leading to the sedimentation and blockage of the fissure channels, thereby hindering the flow of slurry.

## 1. Introduction

Mudstone minerals contain a large amount of clay minerals such as kaolinite, illite and montmorillonite, showing unique engineering characteristics such as water absorption, expansion and argillization [1,2]. For grouting engineering of the fissured mudstone, due to the unique water absorption characteristics of mudstone, a loss of water in grouting slurry will be caused during grouting, thereby accelerating the change in slurry viscosity. Especially for mudstone grouting engineering with complex fissure conditions in the deep, due to the water absorption effect of the mudstone matrix and the superposition of rough fissures, the flow characteristics of the grouting slurry will be seriously affected. If conventional grouting engineering parameters are used, satisfactory grouting results may not be achieved, resulting in waste of manpower and material resources, and even laying greater hidden dangers for safety and stability of the roadways.

The interaction between rocks and water has always been a hot topic of research both domestically and internationally, such as the mechanical properties of water-bearing rocks [3,4,5], the water-rock coupling seepage characteristics [6,7] and the softening mechanism of rocks when encountering water [8,9,10]. Mudstone is a common special soft rock system, and has a certain water absorption ability because its mineral composition contains a large amount of clay minerals such as kaolinite, illite and montmorillonite, and due to capillary phenomenon and the presence of internal fissures in mudstone, the fissured mudstone exhibits strong water absorption [11]. Many scholars have also conducted a series of studies on this issue: HE Man-Chao et al. [12] studied the water absorption characteristics of deep well mudstone with self-developed hydraulic action testers. The study showed that the porosity of mudstone and clay minerals are the main factors affecting the water absorption of mudstone. The more pores in mudstone are and the higher the content of clay minerals is, the greater the water absorption of mudstone will be. Iyare, U. C., et al. [13], conducted triaxial compression tests on mudstone specimens to investigate the influence of water content on the failure behavior. They established experimental models to predict the brittleness, brittle-ductile transition, and ductile failure behavior of water-saturated mudstone. Mudstone with low strength, high porosity, high silica, and clay content exhibited significant weakening in the presence of water. The brittle-ductile transition behavior was influenced by mudstone strength, porosity, and mineralogy. This transition occurred over a wide range of confining pressures, with the threshold under saturated conditions lower than that under dry conditions. The study results indicate that the weak water effect can significantly reduce the depth of the brittle-ductile transition in mudstone. Douma et al. [14]. conducted triaxial deformation tests on mudstone core plugs to investigate the influence of water saturation on the elastic anisotropy of Whitby mudstone in the United Kingdom. Based on stress-strain and ultrasonic velocity data for core plugs at different water saturations under isotropic and anisotropic stress conditions, they estimated the mechanical and physical properties of Whitby mudstone. Patacci et al. [15]. proposed a novel approach to analyze coeval mudstone cap layers based on thickness and composition, providing new insights into gravity flow deposition processes. Bed thickness data from the Castagnola system, derived from high-density turbidites, indicate that overall, turbidites contain more clay compared to mixed-event beds originating from suspension settling.

The flow and grouting effect of slurry in fissures are influenced by multiple factors, such as rock mass, slurry, fissures and grouting technology which all affect the seepage characteristics of grouting slurry. The combined effect of these factors leads to extremely complex flow laws of slurry. Therefore, up to now, there has not been an authoritative theory for grouting in rock fissures. In order to make clear the specific flow situation of slurry inside fissures, domestic and foreign scholars have carried out a series of theoretical derivation of seepage [16,17], and conducted rich indoor grouting simulation research in fissured rock masses [18,19,20]. Hexuan Zhu et al. [21] used CT scanning technology to select single fracture samples with similar three-dimensional fractal dimensions but different dip angles. Subsequently, employing a self-developed single-fracture stress-permeability coupled true triaxial testing system, they conducted permeability tests of grouting materials under different dip angles and different triaxial stress conditions. Sailer et al. [22] explored the issue of compensation grouting in coarse-grained soils, introducing the concept of compensation grouting. The successful implementation of compensation grouting can be demonstrated through subsurface geotechnical deformation measurements, real-time measurements of structures, and the efficiency of the compensation grouting generated as a result. Fraccica et al. [23] mixed sand and silt to form soil covering three orders of magnitude in kilowatts and subjected it to controlled conditions for infiltration using five commonly used binders: two cement-based, two colloidal silica, and one acrylic resin. X-ray tomography scans, unconfined compressive strength tests, and creep tests were employed to assess grout penetration and the performance of the grouted soil. Dong Jia-Bin et al. [24] constructed a three-dimensional digital model to simulate the real rock fissure grid structure, used 3D printing technology to produce corresponding fissure model samples, and further studied the influence of fissure roughness on seepage characteristics through numerical simulation. Li Zheng et al. [25] conducted a study on the law of slurry-water two-phase flow displacement seepage, and obtained the distribution of flow field in rough fissures and the relationship between flow velocity and pressure difference, indicating that the influence of roughness shall be fully considered in the calculation of grouting theory to reasonably determine the grouting pressure.

In summary, regarding the grouting engineering of fissured mudstone, the permeation characteristics of grout slurry under the water absorption effect of fissured mudstone are currently a significant scientific problem that urgently needs to be addressed. Therefore, based on existing theoretical analyses and employing methods such as laboratory experiments, this thesis has conducted research on the permeation characteristics of slurry in water-absorbing rough fissures. This research not only contributes to the improvement of theoretical aspects of rock fissure grouting but also provides guidance for the selection of grouting materials. It holds significant theoretical value and practical significance for the grouting engineering of fissured mudstone.

## 2. Obtaining Mudstone Fissure Parameters and 3D Printing

Due to the complex geological structure morphology of rock masses and their characteristics of concealment and invisibility, it is difficult to directly obtain the geometric distribution information of fissure networks. In this chapter, the CT scanning technology is adopted for fissured mudstone, to obtain the geometric parameters of mudstone fissures through calculation of the fractal dimension, and finally, the physical mold capable of simulating mudstone fissures is prepared through 3D printing technology.

### 2.1. Extraction of the Parameters of Mudstone Fissures

**Figure 1:**
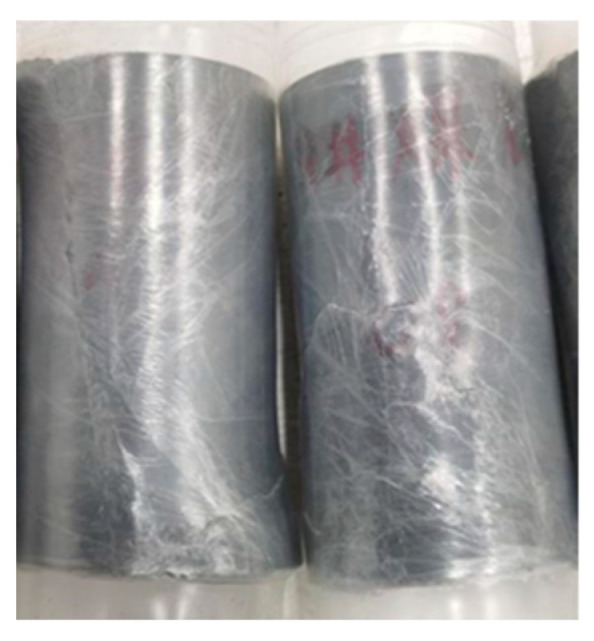
Mudstone sample.

The mudstone test blocks used in the testing are gray mudstone from a certain mine, which is pure and delicate in texture, brittle in nature, with a grayish black color and a natural density of 2.02 g/cm^3^. The specific mudstone sample is as shown in Figure 1. Seven pieces of mudstone with similar wave velocities are taken as test samples to ensure their homogeneity and similar internal fissure characteristics. Three of them are subjected to uniaxial compression testing to determine the peak loads of this group of mudstone, and their average value is selected as the standard for dividing of different stress levels. The average peak value of the four test samples is determined to be 9.88 MPa. Four of the remaining five test samples are loaded with 50%, 70%, 90% and 100% of the average peak value respectively. The fourth test sample is loaded with its own peak stress, and the internal fissure characteristics are detected through CT scanning after loading. The mudstone test sample and the loading condition are shown in Table 1.

**Figure 2:**
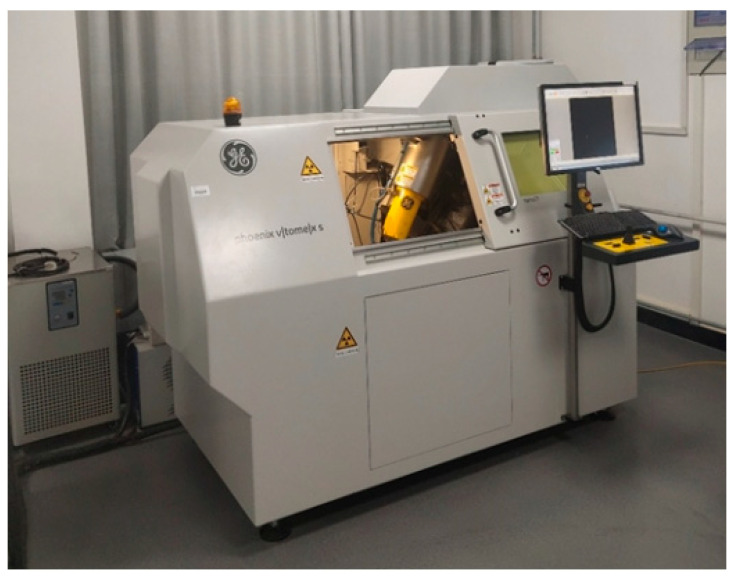
Computed Tomography CT System.

CT scanning is performed on four mudstone blocks 01, 02, 03 and 04 after mechanical testing. For the CT scanning equipment, whose company is Carl Zeiss Industrial from Oberkochen, Germany, the GE phoenix v|tome|x c industrial CT detection system is adopted, as shown in Figure 2. Each mudstone block is scanned in 2000 layers to obtain the original CT image. MATLAB (R2022b) is used to carry out binarization processing to the CT original image, to achieve recognition and extraction of the internal fissures in the mudstone. Finally, the binary image is subjected to background removal processing to eliminate the interference of black background when the fractal dimension is calculated. Firstly, the original image is converted to an 8-bit grayscale image, whose grayscale values range from 0 to 255. When the grayscale value is 0, it represents the region with the lowest grayscale values, and in the original CT image, the cracks in the rock have a grayscale value of 0. Subsequently, a segmentation threshold needs to be set, such that pixels with values greater than the threshold are set to 1, and those below the threshold are set to 0, creating a binary image where pixels are either black or white. This process extracts the cracks, and the resulting image is the binarized version of the original image.

**Figure 3:**
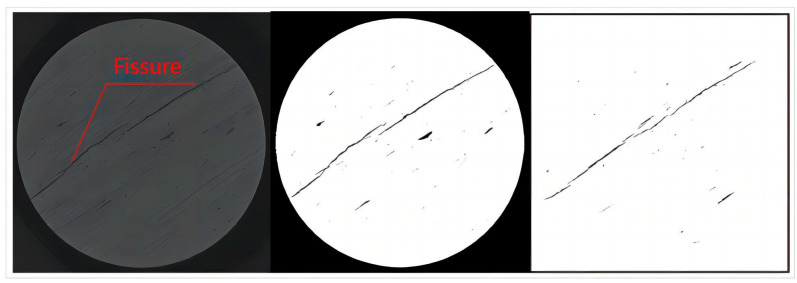
Mudstone CT Image Processing.

The CT slice processing image is shown in Figure 3.

Nowadays, the commonly used fractal dimensions include Hausdorff dimension, information dimension, box counting dimension, correlation dimension, similarity dimension, etc. Each method has its corresponding calculation method. The box counting dimension has intuitive physical significance and a relatively simple calculation method, making it the most widely used method in fractal analysis [26]. The calculation principle of box counting dimension is to evenly divide the space where the fractal image is located into countless boxes, calculate how many boxes are needed to cover the fractal image, change the size of the boxes to observe the changes in the required number of boxes, and finally calculate the box counting dimension of the image through fitting. The calculation formula for box counting dimension is as follows:

(1) $$\dim_{box}(D)=\underset{\varepsilon \to 0}{\lim}\frac{\log N\left(\varepsilon \right)}{\log\left(1/\varepsilon \right)},$$

where: *N*—Number of boxes; *ε*—Side length of the box.

In the image-based binaryzation processing and box-counting dimension method, MATLAB is adopted to fit and calculate the fractal dimensions of the CT images of four mudstone blocks 01, 02, 03, and 04, and the fractal dimensions of the mudstone fissures under different pressure conditions are obtained. Using the Frac-Lab toolbox in MATLAB to fit, calculate the fractal dimensions of the CT images of mudstone specimens numbered 01, 02, 03, and 04.

**Figure 4:**
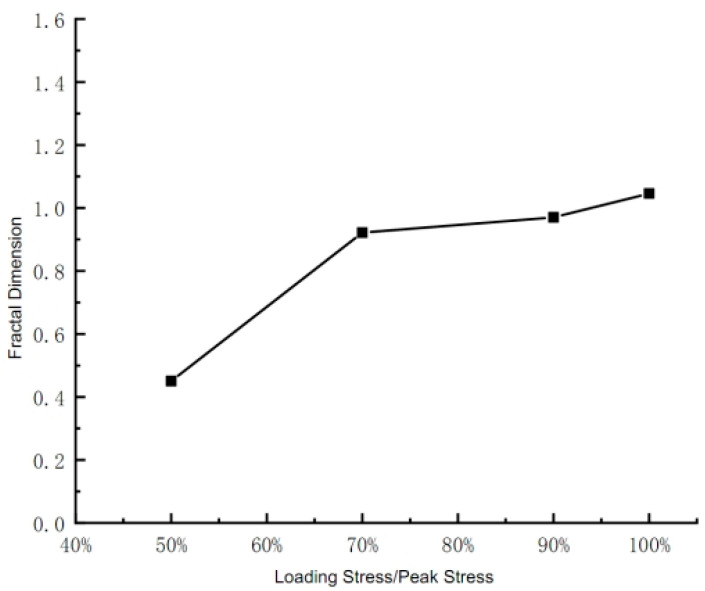
Changes in Fractal Dimension of Mudstone under Different Loading Stresses.

The average fractal dimension of each mudstone block is shown in Table 2, and the relationship between the stress on the mudstone and the average fractal dimension is shown in Figure 4.

As shown in Table 1 and Figure 3, when the axial load on the mudstone is 50% of the peak stress, its average fractal dimension is 0.4504, which is much smaller than the minimum dimension 1 of the normal plane graph. This is because the mudstone has not yet undergone severe failure to produce the main fissures. At this time, the state is the initial stage of the evolution of the initial fissures, with small opening, less distribution, and disconnected original fissures; when the load on the mudstone reaches 70% of the peak stress, its average fractal dimension is 0.922. At this point, the fractal dimension value approaches 1, indicating that the main fissure has basically occurred. Moreover, when the load on the mudstone reaches 50% to 70% of the peak stress, the fractal dimension shows a sharp increase, indicating that a more severe failure of the mudstone will occur at this stage, which is precisely due to this failure that the main fissure has been generated; when the load reaches 90% and the peak value, the average fractal dimensions are 0.9703 and 1.046 respectively, indicating that the main fissure is gradually developing with the increase of load until the mudstone fails. Considering the practical significance of the plane fractal dimension and the preparation of the fissure mold, in this paper, the fissure parameter with a fractal dimension greater than 1 will be selected, that is, the fissure fractal dimension 1.046 when the mudstone reaches the peak value is taken as the parameter for production of the fissure mold.

### 2.2. Preparation of Rough Fissure Molds

**Figure 5:**
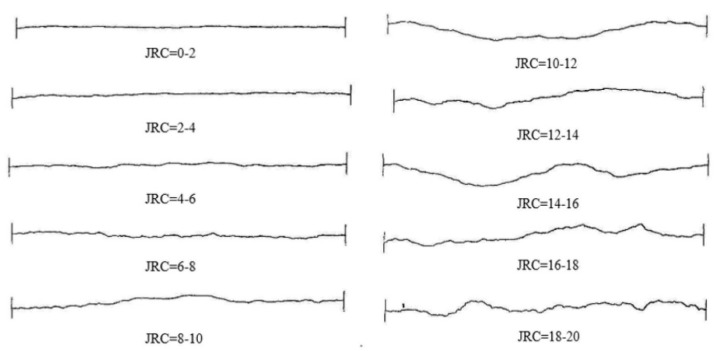
Ten Barton Standard Roughness Curves.

At present, the JRC roughness coefficient is a commonly used parameter to represent the tortuous roughness of the fissure face. The most widely used JRC curve is the Barton curve. In 1977, Barton provided a total of 10 standard JRC profile lines based on a large number of tests [27], as shown in Figure 5.

At the same time, there is also a good correspondence between the JRC value and the fractal dimension. Academician XIE He-Ping [28] proposed a fractal model for joints based on previous research, and found that the fractal dimension D has the following relationship with the JRC value:

(2) $$JRC=85.6721{\left(D-1\right)}^{0.5679},$$

**Figure 6:**
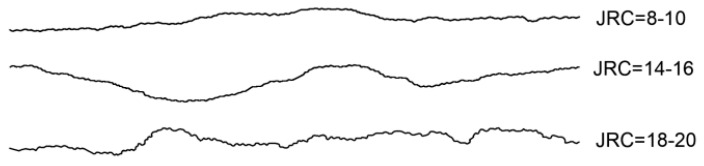
Three Roughness Curves.

**Figure 7:**
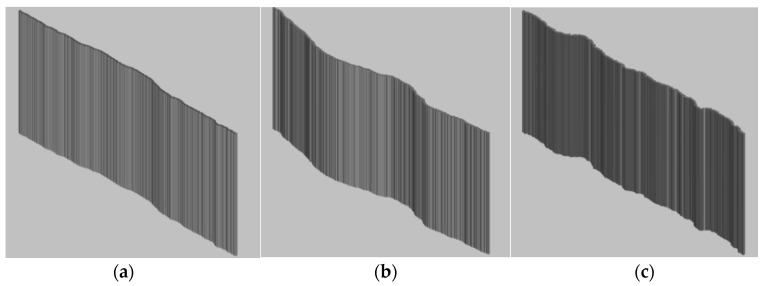
3D Models of Three Roughness Levels; (**a**) JRC = 8–10; (**b**) JRC = 14–16;(**c**) JRC = 18–20.

From the previous section, it can be seen that the fractal dimension of the fissures generated inside the mudstone is 1.046 when loaded to the peak stress. By substituting the fractal dimension value into Equation (2), the corresponding JRC value at this time is 14.908. With this value as a reference, three roughness curves, JRC = 8–10, JRC = 14–16 and JRC = 18–20, are ultimately selected as prototypes for the production of rough fissure templates. These three Barton standard curves are extracted into Auto CAD, to draw and reconstruct three roughness curves of JRC = 8–10, 14–16 and 18–20. The specific curves are shown in Figure 6. Based on these three roughness curves, a 3D model with a length of 300 mm, a width of 120 mm and a thickness of 2 mm is produced, as shown in Figure 7.

**Figure 8:**
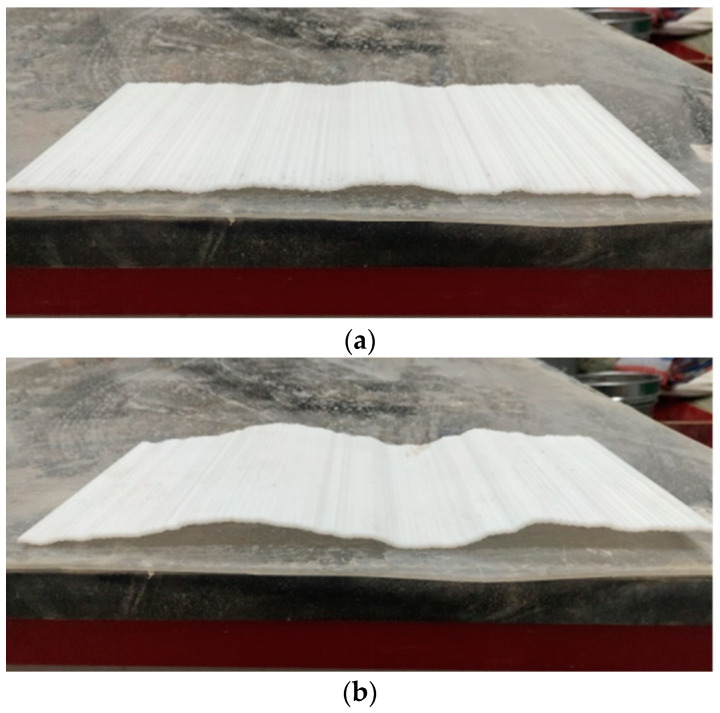

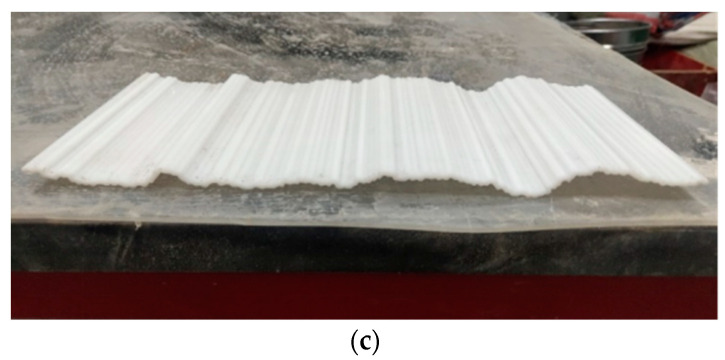
Physical Models of Fissures with Different Roughness Levels; (**a**) Fissure Mold for JRC of 8–10; (**b**) Rough Fissure Mold for JRC of 14–16; (**c**) Rough Fissure Mold for JRC of 18–20.

These three models are uploaded to 3D printer for processing and production. White resin is adopted as the material. This material has the characteristics of light weight, ageing resistance, high toughness, imperviousness, easy cleaning, etc. and is widely used in 3D printing technology. The molding technology produced is relatively mature, and can meet the requirements of rough fissure tortuosity. The specific completed physical model of fissures is shown in Figure 8.

## 3. Development of Materials with the Same Water Absorption as the Mudstone

Before conduction of the rough single fissured mudstone grouting testing, it is necessary to obtain test blocks of fissured mudstone with appropriate size and complete shape. However, it is usually difficult to obtain natural underground mudstone samples, and obtaining of the required test blocks of fissured mudstone through on-site sampling is also difficult. In response to this issue, in this paper, similar materials are used to replace mudstone for subsequent grouting testing.

### 3.1. Mudstone Water Absorption Testing

**Figure 9:**
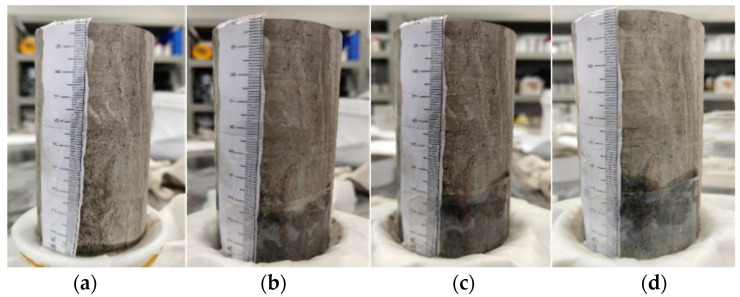

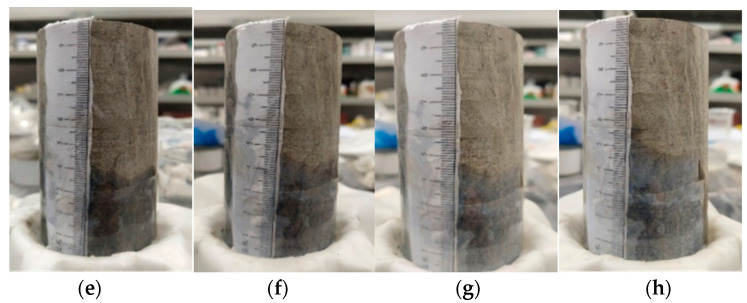
Process of Water Absorption Testing of Mudstone Test Blocks; (**a**) 1 min; (**b**) 5 min; (**c**) 10 min; (**d**) 20 min; (**e**) 40 min; (**f**) 60 min; (**g**) 90 min; (**h**) 120 min.

To ensure that the water loss effect of the slurry in the subsequent flow in the fissure is similar to that of the actual mudstone, it is necessary to select an appropriate ratio of similar materials through mudstone water absorption testing. This water absorption testing mainly focuses on the permeability characteristics of mudstone in terms of the permeation of water in slurry. The main principle of this testing is that mudstone automatically absorbs water into the core without external force. Specific testing method: The filter paper is fixed at the mouth of a beaker fully filled with water, and the dried mudstone is put on the filter paper. During the process of water absorption, record the water infiltration height using photos, and quantitatively obtain the water absorption of the mudstone using the weighing method. The specific process of the water absorption testing of mudstone is shown in Figure 9.

The water infiltration height can indirectly indicate the water absorption of mudstone. The specific variation pattern of the water infiltration height can be observed from Figure 8, the diagram of the water absorption test process for mudstone specimens. The water infiltration height gradually increases with the passage of time, and at 120 min, it is evident that it has not reached the limit of water absorption for this block of mudstone. However, since the normal grouting process time is usually not excessively long, this water absorption test considers the process only up to 120 min.

**Figure 10:**
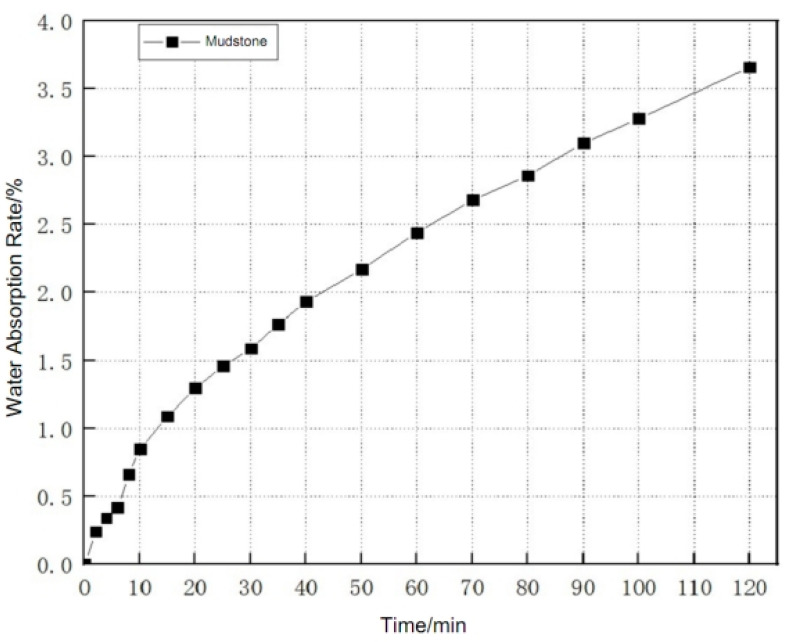
Characteristic Curve of Water Absorption of Mudstone.

The water absorption data of mudstone at different times are recorded, and processed through the Origin (2022 SR1) analysis software. The water absorption rate and time curve is drawn. The water absorption rate is the ratio of the mass of water absorbed in mudstone to the dry mass of mudstone, and the obtained water absorption characteristic image of mudstone is shown in Figure 10.

(3) $$W=\frac{B-G}{G}\times 100\%,$$

where: *W*—Water absorption rate of mudstone, in %;

*B*—Mass of mudstone containing water, in g;

*G*—Mass of mudstone after drying, in g.

### 3.2. Ratio of Similar Materials for Mudstone

The grouting test mainly requires the physical properties of water of similar materials, that is, the properties of materials with a rate of water absorption similar to that of natural mudstone, so as to simulate the water loss effect of grouting material seepage in rough fissured mudstone. In the ratio of similar materials, the sand-adhesive mass ratio and water-gypsum mass ratio are the main control factors. The sand-adhesive mass ratio is selected as three horizontal factors: 2:1, 5:1 and 8:1, while the water-gypsum mass ratio is selected as three testing levels: 0, 0.5 and 1. The water consumption is all 1/10 of the total solid mass. The specific ratio of the similar materials is shown in Table 3.

**Figure 11:**
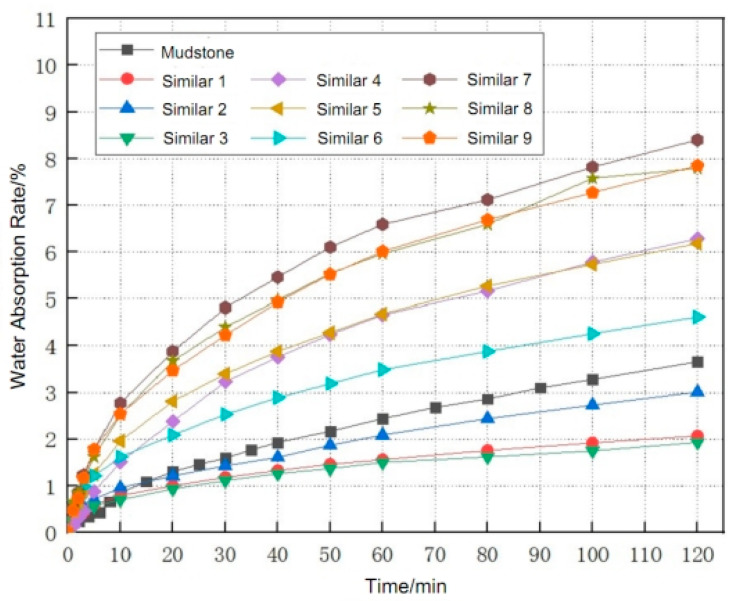
Water Absorption Characteristic Curves of Mudstone and Similar Materials with Different Ratios.

The above water absorption testing is carried out to the above nine groups of similar materials. In order to reduce the testing error, two test blocks are arranged for each group, and the final water absorption result is the average water absorption of the two test blocks. The water absorption curves of the nine groups of similar materials with different ratios are compared with the water absorption curves of mudstone, to obtain the best similar material ratio for production of rough fissured mudstone grouting test blocks. The comparison image of the water absorption between similar materials with different ratios and mudstone is shown in Figure 11.

From Figure 11, it can be clearly seen that the trend and values of the water absorption characteristic curves of the second group with similar ratio are basically the same as those of the actual mudstone. Therefore, this group of ratio is selected as a similar ratio for the rough single fissured mudstone grouting test block, with cement:gypsum:quartz sand of 1:2:6, and water consumption of 1/10 of the solid mass.

## 4. Seepage Testing of Rough Fissured Water Absorption Mudstone

### 4.1. Preparation of Rough Single Fissured Grouting Test Blocks

**Figure 12:**
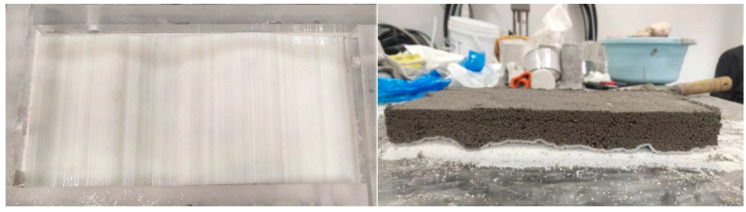
Placement of Roughness Mold and Laying of Test Blocks.

**Figure 13:**
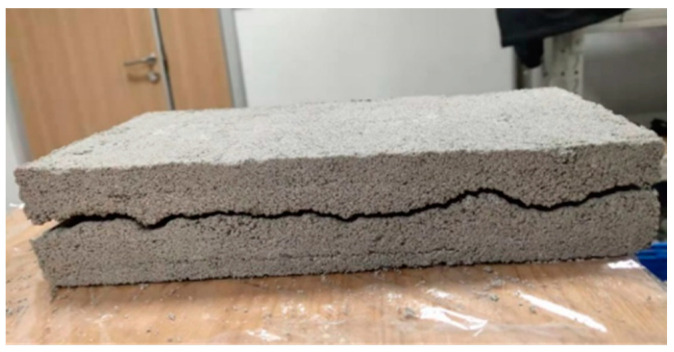
Preparation of Rough Single Fissured Grouting Preliminary Test Block at JRC of 18–20.

The appearance dimensions of the rough single fissured grouting test block are designed to be 300 × 120 × 60 mm, and the fissure opening is 2 mm. The specific production method is illustrated by preparing a rough single fissured grouting test block with a roughness JRC of 18–20. The steps are shown as follows:

(1) The Portland cement, gypsum, quartz sand and water are weighed with electronic scales, beakers, etc. as per the ratio of similar materials for rough fissured grouting test blocks.
(2) The weighed cement, gypsum and quartz sand are put into a stirrer to mix uniformly, with a stirring time of about 2 min.
(3) The weighed water is poured into the uniformly stirred solid material in the previous step and stirred thoroughly, with a stirring time of still about 2 min.
(4) The mold with a roughness level at JRC of 18–20 is placed on the tabletop, and a layer of lubricating oil is applied on the surface of the mold. The stirred similar material is laid on the mold with this roughness level, with the aim of producing a test block with a roughness level of the rough face at JRC of 18–20. To prevent deformation of the roughness mold during laying, an appropriate amount of quartz sand is laid under the mold for bedding. The placing position of the fissure mold and the laid test block 1 are shown in Figure 12.
(5) After the production of the test block 1 is completed, it is cured at room temperature for 24 h. The roughness mold is removed, and the roughness mold is turned over before next laying. The above steps are repeated to produce the test block 2.
(6) After the test block 1 and test block 2 are cured together for 7 days, the two test blocks are spliced, and the two test blocks are padded with a 2 mm high gasket and fixed. The preparation of the rough single fissured grouting preliminary test blocks with a fissure opening of 2 mm and JRC of 18–20 is completed, as shown in Figure 13.

**Figure 14:**
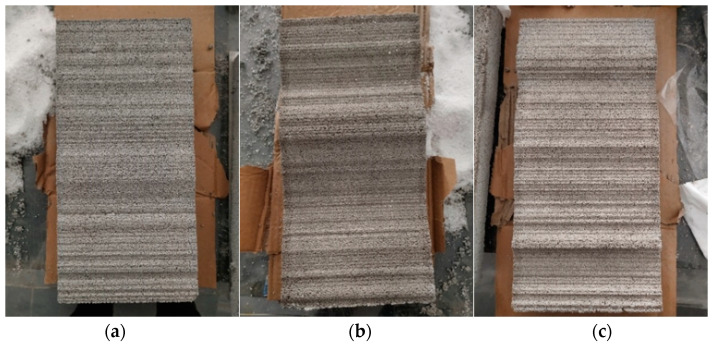
Fissure Face of Rough Single Fissured Grouting Test Block; (**a**) JRC = 8–10; (**b**) JRC = 14–16; (**c**) JRC = 18–20.

As per the above steps, the rough single fissured grouting test blocks with three different roughness levels can be obtained, and the rough fissure faces of the test blocks are shown in Figure 14. It can be observed that the fissures inside the test blocks are relatively complete, which can better meet the needs of different roughness levels in subsequent testing. At the same time, after the preparation of the rough single fissured grouting preliminary test blocks with three roughness levels is completed, it is necessary to seal the fissures on the both sides of the test blocks with an electric gluing gun to prevent slurry from flowing out from the both sides during the subsequent grouting process, so that the rough single fissured grouting test blocks meet the requirements for the grouting testing.

### 4.2. Rough Single Fissured Grouting Simulation Testing

In order to reveal the water loss effect of grouting slurry flowing in the rough fissures of mudstone, the simulation testing of rough single fissured grouting is conducted. When the testing plan is designed, it shall be noted that in actual grouting engineering, as the grouting distance and grouting time increase, the grouting stress has sharply decreased when the actual slurry reaches the interior. Therefore, in this chapter, the grouting stress is not considered, and the research focuses the seepage effect of mudstone on water in stable flowing water slurry and the sedimentation effect of rough fissures on the slurry, to simulate the water loss phenomenon of cement slurry during the grouting process of rough fissured mudstone.

**Figure 15:**
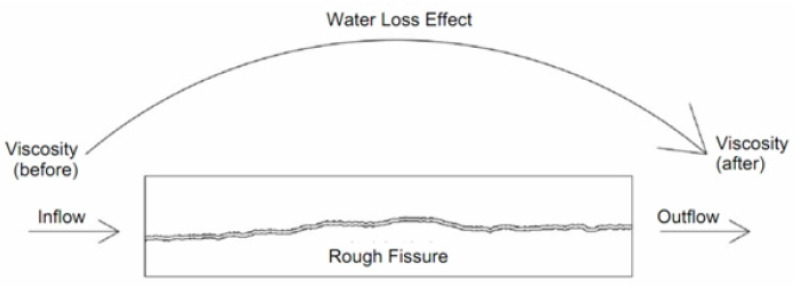
Schematic Diagram of Simulation Testing for Water Loss Grouting in Rough Fissures.

Per the design and requirements for the grouting simulation testing in this chapter, grouting test blocks of mudstone and similar materials with three roughness levels at JRC = 8–10, JRC = 14–16 and JRC = 18–20 are prepared. The fissure opening of each rough single fissured grouting test block is 2 mm. In this grouting testing, the water loss effect of the grouting slurry is indirectly represented through the viscosity change of the slurry. By measuring the viscosity of the slurry before and after the testing, the slurry after grouting is collected every 20 s and stirred appropriately. After stirring, the viscosity after grouting is immediately measured with a rotary viscometer. The collection and measurement are repeated for three times, and the data is recorded. Schematic diagram of simulation testing for water loss grouting in rough fissures is shown as Figure 15.

### 4.3. Grouting Testing Results and Analysis

This testing focuses on the study of the roughness of mudstone fissures and the influence of water absorption on the permeability of grouting slurry, where the permeability is represented through the viscosity of the slurry.

The ZNN-D6X six-speed rotary viscometer, from the company KENCE Instrument (Shanghai, China), has six rotating speeds: 3, 6, 100, 200, 300 and 600 r/min respectively. Generally, as per the definition of apparent viscosity, the apparent viscosity at a certain shear rate can be represented through the following formula:

(4) $$\mu_{\alpha }=\frac{\tau }{\gamma }=(\frac{0.511\theta_{N}}{1.703N})=\frac{0.3\theta_{N}}{N}=k\theta_{N},$$

where: *μ_α_*—Apparent viscosity, in pa·s;

*N*—Rotating speed, in r/min;

*θ_N_*—Dial reading at the rotating speed of *N*;

*k*—Conversion coefficient.

Through the Formula (4), the dial reading measured at any shear rate, that is, at any rotating speed of the rotor, can be converted into apparent viscosity. The conversion coefficient k for the commonly used six rotating speeds is shown in Table 4.

Usually, when the viscosity of the slurry is measured, in order to facilitate comparison, the apparent viscosity at 600 r/min is usually measured, and the calculation formula for the measured slurry viscosity is shown as follows:

(5) $$\mu =0.5\theta_{600}\times 10^{-3}$$

**Figure 16:**
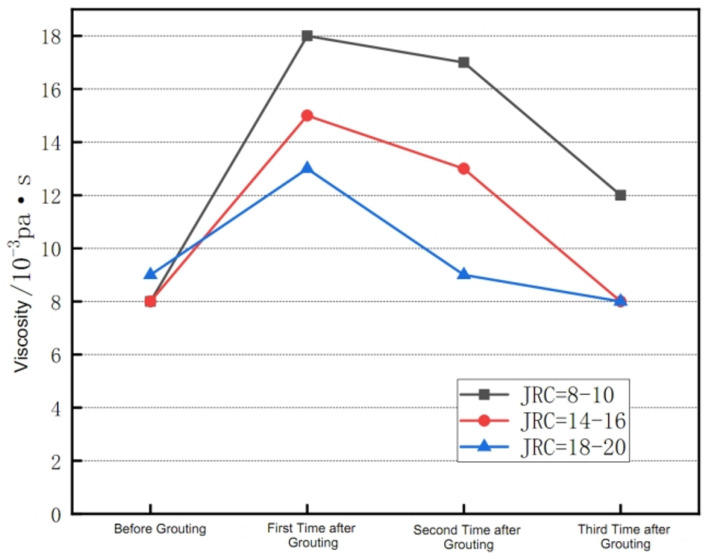
Changes in Viscosity of Fissure Grouting Slurry for Water Absorption Mudstone with Different roughness levels.

The final results of the single fissured grouting testing with different roughness levels are shown in Figure 16.

From Figure 16, it can be seen that the grouting slurry has the following characteristics when passing through fissures of the water absorbing mudstone with different roughness levels:

(1) When the grouting slurry passes through the fissures with the roughness level at JRC of 8–10, the viscosity measured for the first time after grouting has significantly increased compared with the viscosity of the slurry before grouting. Due to the short flow time of the slurry at this time, the influence of the time-variant viscosity can be basically ruled out. Therefore, the main factor for drastic change in viscosity of the grouting slurry shall be the water absorption properties of the grouted material itself.
(2) The results of the second and third viscosity measurement in grouting testing of the fissures with the roughness level at JRC of 8–10 show a gradual decreasing trend, indicating that the influence of the water absorption on the subsequent grouting slurry is weakened. This may be due to the rough fissured channel experiencing the grouting slurry flow, and its weakened water absorption after the surfaces of the both sides of the fissure enter a wet state because of water absorption. At the same time, in consideration of faster flow speed of the slurry, the fissure face cannot fully absorb water from the slurry, resulting in a weakened water loss effect on the subsequent slurry under the combined action of the both.
(3) When the grouting slurry passes through the fissures with a roughness level at JRC of 14–16, the viscosity value measured for the first time after grouting still increases, indicating that the water absorption properties of the mudstone still play a role. The viscosity value is smaller compared with that in the first time at JRC of 8–10, and the viscosity measured for the second and third times is slightly higher than that of the slurry before grouting. The change trend and conclusions in the grouting testing of fissures with the roughness level at JRC of 18–20 are basically similar to those for the fissures with the roughness level at JRC of 14–16, and the viscosity measured in the second and third times is basically the same as that of the slurry viscosity before grouting. These two groups of tests have shown that the higher the roughness of the fissures is, the weaker the water loss effect on the subsequent grouting slurry will be. Therefore, a supplementary testing will be conducted to investigate the reasons for the conclusions.

### 4.4. Supplementary Testing Results and Analysis of Rough Single Fissured Grouting

**Figure 17:**
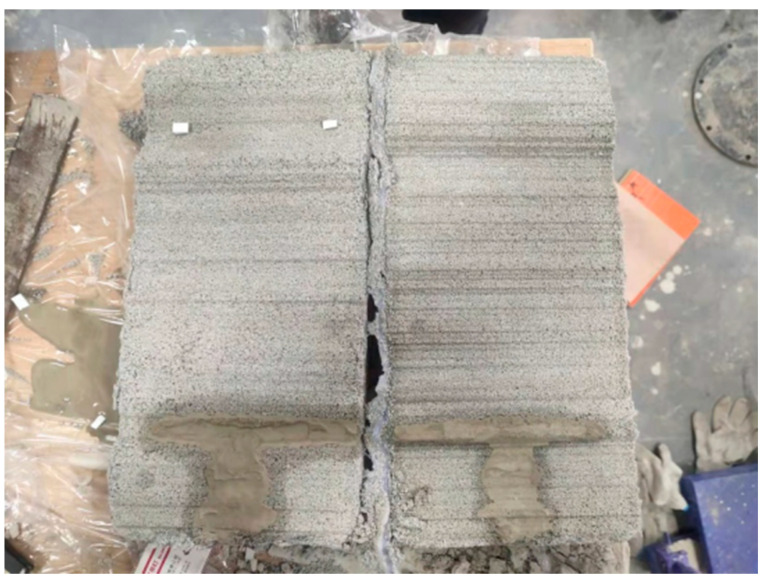
Distribution of Grouting Slurry in Rough Fissures.

**Figure 18:**
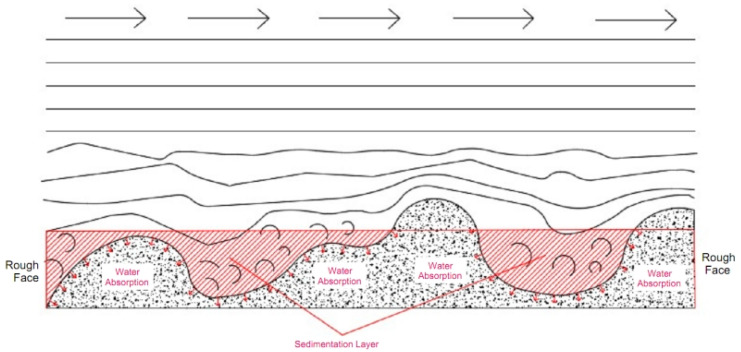
Schematic Diagram of Seepage and Water Loss in Grouting Slurry in Rough Fissures.

In order to probe into the reasons of the testing conclusion 3, the test blocks with roughness level at JRC of 18–20 are remade for additional supplementary testing. Due to the large flow rate of the pneumatic pump grouting compared with the size of the grouting test blocks, it is difficult to control the grouting and too high flow rate of the slurry through the fissures, which makes it difficult to accurately obtain the corresponding testing phenomenon. Therefore, for the grouting device in this additional testing, the 500 mL capacity syringe that is easy to control is adopted. The specific testing process, phenomena, and results are shown as follows:

When the grouting slurry is injected into the rough fissures at JRC of 18–20 with a syringe, the injection cannot proceed after the slurry of about 150 mL is injected. Forced grouting can cause the slurry to spray out from the grouting port. At this time, the grouting test block is opened to observe the distribution of slurry on the fissure face, as shown in Figure 17:

From Figure 17, it can be seen that the distribution of grouting slurry is T-shaped, and at this time, there is blockage in the fissures. After analysis, there are two main reasons for this phenomenon: firstly, the slurry is affected by a large rough wall during the flow process, which hinders the flow of the slurry; secondly the grouting stress and volume of the syringe are too small, resulting in a low flow rate of the slurry entering the fissures, to provide sufficient water absorption conditions for the rough fissure face, leading to rapid solidification of the slurry and hindering the subsequent grouting process. For these two reasons, it can be analyzed in detail why the water loss effect on the subsequent grouting of slurry decreases with the increase of roughness level of the fissure, as shown in Figure 18.

As shown in Figure 18, the slurry mainly relies on the inertial force provided from the grouting stress and the driving force of the subsequent fluid during the flowing process, while the obstruction effect mainly comes from the cohesive force of the slurry itself and the frictional resistance between the slurry and the rock wall. For fissures with larger roughness, the presence of more roughness elements will also prevent the flow of the slurry. When the slurry encounters larger roughness elements, the flow direction of partial fluid will change and no longer move forward. This part of the slurry that no longer flows forward will be affected by the water absorption properties of the mudstone, accelerating the solidification of this part of the slurry. An obstruction effect caused due to the roughness elements of the rough face is created, and a layer of solidified slurry sedimentation is formed on the fissure face, which isolates the subsequent slurry from the contact with the fissure surface, thereby reducing the water loss effect of the subsequent slurry.

This theory can also explain that when compared with fissures with higher roughness, the viscosity value of the slurry passing through fissures with lower roughness is higher. This is mainly because when the slurry passes through fissures with higher roughness, due to the obstruction of roughness elements in the rough face, a layer of slurry in contact with the fissure surface undergoes a water loss effect. The slurry passing through the fissure outlet is basically the slurry in the middle part that is less affected due to the water loss effect. Therefore, in the measurement results, it is indicated that the viscosity of the slurry passing through the fissures with high roughness is smaller. In fact, it is the fissures with high roughness that retain the water loss part of the slurry inside the fissures, resulting in this result.

Through the above testing results and analysis, the characteristics of viscosity changes of the rough fissured grouting slurry of water absorbing mudstone during the grouting process can be obtained, thus indirectly representing the changes in the permeability performance of the slurry:

(1) When the roughness level of the fissures in water absorbing mudstone is small, the main factor affecting the viscosity change of the slurry is the water absorption of the mudstone. This property causes the water loss of slurry, which affects the permeability of the slurry. However, for the part of the fissure where the slurry has already flowed, the fissure face enters a relatively wet state due to water absorption. If the slurry is kept in a flowing state at this time, the water loss effect of the subsequent grouting slurry will be weakened.
(2) When the roughness level of the fissures in water absorbing mudstone is large, due to the presence of many roughness elements on the rough face, the grouting slurry needs more energy to overcome the obstruction of the roughness elements in the early stage. At the same time, under the joint influence of the roughness elements and water absorption, sedimentation areas will quickly form, causing the fissure channel to narrow. The permeability of the slurry injected in the later stage is not significantly affected due to the water absorption of mudstone, but the rapid accumulation of sedimentary areas will lead to narrower fissure opening, smaller grouting flow rate, and even blockage in severe cases, indirectly affecting the permeability of the slurry.

## 5. Conclusions

In this paper, based on the CT scanning and fractal theory, the geometric parameters of the mudstone fissures are obtained, the physical model of single fissured channel is obtained in combination with Barton standard curves and 3D printing technology, and similar materials of mudstone are developed based on the water absorption of natural mudstone to prepare single fissured water-absorbing grouting test blocks with different roughness levels for the grouting simulation testing. By analyzing the viscosity change characteristics of the grouting slurry before and after grouting, the seepage characteristics of the grouting slurry in the rough fissures of the water-absorbing mudstone are revealed, and the conclusions are shown as follows:

(1) The fractal dimension of fissures in the mudstone test blocks is calculated through CT scanning and fractal theory. The average fractal dimensions corresponding to mudstone fissures with peak stresses of 50%, 70%, 90% and 100% as the loading stresses are 0.4504, 0.922, 0.9703 and 1.046 respectively. There is a clear positive correlation between axial stress and the fractal dimension of the cracks. The analysis indicates that the strength of the mudstone when producing the main fissure should be between 50% and 70% of the peak strength.
(2) 1.046 is selected as the parameter for production of the fissure mold, and the corresponding JRC of 14.908 is obtained through the conversion formula between fractal dimension and roughness level JRC. Three roughness level curves in the Barton standard curves JRC = 8–10, 14–16 and 18–20 are selected as the prototype of the rough fissure, and fissure molds with different roughness levels are produced through model establishment and 3D printing.
(3) Based on the water absorption properties of mudstone, the spontaneous absorption testing is conducted on natural mudstone, the water absorption rate and time curve of mudstone is obtained, and a ratio for the materials with the similar water absorption properties of actual mudstone is achieved by comparing and analyzing the water absorption rate and time curve of similar materials with 9 ratios. The specific ratio is cement: gypsum: quartz sand of 1:2:6, and the water consumption is 1/10 of the solid mass.
(4) For water absorbing rough fissures, when the roughness level is small, the grouting slurry will have a significant water loss effect after passing through the mudstone fissures. However, as the slurry flows, the water loss effect of the subsequent grouting slurry will weaken. For the fissures with a high roughness level, the grouting slurry will mainly deposit due to the tortuosity of the rough face, showing a weak water loss effect of the outflow slurry. The actual sedimentation layer has already experienced a strong water loss effect, and the rapid accumulation of the sedimentation layer will cause the fissure opening to narrow, thereby hindering the flow of the grouting slurry.
(5) When the cracks in the mudstone in the grouting area are not fully developed, it is advisable to increase the water-cement ratio of the slurry appropriately and enhance the grouting stress. This helps in rapidly moistening the crack surfaces while ensuring that the slurry has a higher flow rate, thus more quickly reducing the water loss effect of the slurry. When the degree of crack development is higher, the combined action of the rough surfaces of the cracks and their water-absorbing nature will lead to deposition and blockage of the crack channels. At this point, it is recommended to use ultrafine cement and additives such as retarders to mitigate the narrowing of cracks caused by their roughness and the impact on the water absorption properties of the rock.

## Figures and Tables

**Table 1:** Mudstone Test Samples and Loading Conditions.

Test Sample No.	Diameter of Test Sample/mm	Height of Test Sample/mm	Mass of Test Sample/g	Loading Stress/MPa
01	49.48	99.84	382.5	4.94 (50% of Peak Value)
02	50.17	99.81	397	6.92 (70% of Peak Value)
03	50.14	100.39	409.9	8.89 (90% of Peak Value)
04	50.07	100.39	411.5	Loaded to the peak stress

**Table 2:** Average Fractal Dimension of Mudstone under Different Axial Stresses.

Test Sample No.	01 (50% of Peak Strength)	02 (70% of Peak Value)	03 (90% of Peak Strength)	04 (Peak Strength Reached)
Average Fractal Dimension	0.4504	0.922	0.9703	1.046

**Table 3:** Ratio Scheme of Similar Materials.

Testing No.	Cement Mass Ratio	Gypsum Mass Ratio	Quartz Sand Mass Ratio	Water
1	0	1	2	The water consumption is 1/10 of the total solid mass.
2	1	2	6	
3	1	1	4	
4	0	1	5	
5	1	2	15	
6	1	1	10	
7	0	1	8	
8	1	2	24	
9	1	1	16	

**Table 4:** Conversion Coefficient for Six-Speed Rotary Viscometer.

Rotating Speed/r·min^−1^	3	6	100	200	300	600
Viscosity Conversion Coefficient/10^−3^	100	50	3	1.5	1	0.5

## Data Availability

Data are contained within the article.

## References

[1] He M.C. (2014). Progress and challenges of soft rock engineering in depth. J. China Coal Soc..

[2] Döner Z., Hu Q., Kumral M., Kibria M.G., Qiao H., Sun M. (2021). Petrophysical characteristics of Silurian mudstones from central Taurides in southern Turkey. J. Earth Sci..

[3] Obasi I.A., Ahmed J.B., Anakwuba E.K., Aigbadon G.O., Akudo E.O., Onwa N.M. (2023). Assessment of aquifer vulnerability in fractured rocks in the Abakaliki area, southeastern Nigeria, using geophysical and geological data. Environ. Earth Sci..

[4] Jiang L.S., Zhao D.K., Zhou J.P. (2021). Study on the Acoustic Emission Characteristics of Shale with Different Moisture Content under Uniaxial Compressive Tests. Chin. J. Undergr. Space Eng..

[5] Terrone M., Paliaga G., Bazzurro N., Marchese A., Faccini F. (2021). Groundwater resources in a fractured-rock aquifer, Conglomerate of Portofino. J. Maps.

[6] Ghadrdan M., Shaghaghi T., Tolooiyan A. (2020). Effect of negative excess pore-water pressure on the stability of excavated slopes. J. Géotechnique Lett..

[7] di Lernia A., Cotecchia F., Elia G., Tagarelli V., Santaloia F., Palladino G. (2022). Assessing the influence of the hydraulic boundary conditions on clay slope stability: The Fontana Monte case study. Eng. Geol..

[8] Papeschi S., Mazzarini F., Musumeci G., Cruden A.R. (2022). Emplacement of a felsic dyke swarm during progressive heterogeneous deformation, Eastern Elba Dyke Complex (Island of Elba, Italy). J. Struct. Geol..

[9] Julia F., Vladimir L., Sergey R., David Z. (2014). Effects of hydrothermal alterations on physical and mechanical properties of rocks in the Kuril–Kamchatka island arc. Eng. Geol..

[10] Sun M., Yu J., Wu X., Ding Y., Fu T., Yang Y., Jiang J. (2021). Mechanical Behavior of Weathered Granite Exposed to Water. Appl. Sci..

[11] Huang K., Yu F., Zhang W., Tong K., Guo J., Li S., Chen S., Dai Z. (2023). Relationship between capillary water absorption mechanism and pore structure and microfracture of red-layer mudstone in central Sichuan. Bull. Eng. Geol. Environ..

[12] He M.C., Zhou L., Li D.J. (2008). Experimental research on hydrophilic characteristics of mudstone in deep well. Chin. J. Rock Mech. Eng..

[13] Iyare U.C., Blake O.O., Frash L.P., Carey J.W., Jones D., Ramjarrie K. (2023). Water-Weakening Effects on the Failure Behavior of Mudstones. Rock Mech. Rock Eng..

[14] Douma L.A., Dautriat J., Sarout J., Dewhurst D.N., Barnhoorn A. (2020). Impact of water saturation on the elastic anisotropy of the Whitby Mudstone, United Kingdom. Geophysics.

[15] Patacci M., Marini M., Felletti F., Di Giulio A., Setti M., McCaffrey W. (2020). Origin of mud in turbidites and hybrid event beds: Insight from ponded mudstone caps of the Castagnola turbidite system (north-west Italy). J. Sedimentol..

[16] Coupette F., Schilling T. (2022). Exactly solvable percolation problems. J. Phys. Rev. E.

[17] Baker C. (1974). Comments on paper rock stabilization in rock mechanics. J. Muler.

[18] Mohammadzamani D., Lavasan A.A., Wichtmann T. (2023). Tail void grouting material: A parametric study on the role of hydro-mechanical characteristics in mechanized tunneling. Tunn. Undergr. Space Technol..

[19] Gangrade R.M., Grasmick J.G., Mooney M.A. (2022). Probabilistic assessment of void risk and grouting volume for tunneling applications. Rock Mech. Rock Eng..

[20] Hassler L., Stille H., Hakansson U. (1987). Simulation of grouting in jointed rock. Proceedings of the 6th ISRM Congress.

[21] Zhu H., Han L. (2022). Experimental Study on Grouting Seepage Characteristics of Single-Fractured Rock Masses with Different Inclination Angles under Three-Dimensional Stress. Geofluids.

[22] Sailer M., Fillibeck J., Geuder S. (2022). A new approach for compensation grouting in highly permeable gravel. Geotechnical Aspects of Underground Construction in Soft Ground.

[23] Fraccica A., Spagnoli G., Romero E., Arroyo M., Gómez R. (2022). Permeation grouting of silt-sand mixtures. Transp. Geotech..

[24] Dong J.B. (2020). LBM Simulations and Experimental Validations of Fluid Flow through Single Fractures in Rock Media.

[25] Li Z. (2019). Study on Seepage and Diffusion of Rough Rock Fracture Based on Fractal Theory.

[26] Yin Y., Ren Q., Lei S., Zhou J., Xu L., Wang T. (2024). Mesoscopic crack pattern fractal dimension-based concrete damage identification. Eng. Fract. Mech..

[27] Barton N., Choubey V. (1977). The shear strength of rock joints in theory and practice. Rock Mech..

[28] Xie H. (1995). Fractal description of rock joints. Chin. J. Geotech. Eng..

